# Safety, immunogenicity, and lot-to-lot consistency of a split-virion quadrivalent influenza vaccine in younger and older adults: A phase III randomized, double-blind clinical trial

**DOI:** 10.1080/21645515.2017.1384106

**Published:** 2017-11-27

**Authors:** Sanie Sesay, Jerzy Brzostek, Ingo Meyer, Yves Donazzolo, Geert Leroux-Roels, Régine Rouzier, Béatrice Astruc, Henryk Szymanski, Nicole Toursarkissian, Corinne Vandermeulen, Edyta Kowalska, Pierre Van Damme, Camille Salamand, Stephanie Pepin

**Affiliations:** a Sanofi Pasteur, Marcy l'Etoile, France; b Clinic of Infectious Diseases Health Care Team, Dębica, Poland; c CRS Clinical Research Services Kiel GmbH, Lübeck, Germany; d Eurofins-Optimed, Gières, France; e Center for Vaccinology, Ghent University and University Hospital, Gent, Belgium; f Centre CAP, Centre Médical Odysseum, Montpellier, France; g Biotrial, Rennes Cedex, France; h St Hedwig of Silesia Hospital, Department of Paediatrics, Prusicka, Trzebnica, Poland; i Medic Trials, Berlin, Germany; j Leuven University Vaccinology Center, Leuven, Belgium; k “Medyk”, Wola, Poland; l Centre for the Evaluation of Vaccination, Vaccine & Infectious Disease Institute, Antwerpen (Wilrijk), Belgium

**Keywords:** adult, elderly, immunogenicity, inactivated influenza vaccine, randomized controlled trial, safety, quadrivalent influenza vaccine

## Abstract

Here, we report a randomized multicenter phase III trial assessing the lot-to-lot consistency of the 2014–2015 Northern Hemisphere quadrivalent split-virion inactivated influenza vaccine (IIV4; Sanofi Pasteur) and comparing its immunogenicity and safety with that of trivalent inactivated influenza vaccine (IIV3) in younger and older adults (EudraCT no. 2014-000785-21). Younger (18–60 y, n = 1114) and older (>60 y, n = 1111) adults were randomized 2:2:2:1:1 to receive a single dose of one of three lots of IIV4, the licensed IIV3 containing the B Yamagata lineage strain, or an investigational IIV3 containing the B Victoria lineage strain. Post-vaccination (day 21) hemagglutination inhibition antibody titers were equivalent for the three IIV4 lots. For the pooled IIV4s vs. IIV3, hemagglutination inhibition antibody titers were also non-inferior for the A strains, non-inferior for the B strain when present in the comparator IIV3, and superior for the B strain lineage when absent from the comparator IIV3. For all vaccine strains, seroprotection rates were ≥98% in younger adults and ≥90% in older adults. IIV4 also increased seroneutralizing antibody titers against all three vaccine strains of influenza. All vaccines were well tolerated, with no safety concerns identified. Solicited injection-site reactions were similar for IIV4 and IIV3 and mostly grade 1 and transient. This study showed that in younger and older adults, IIV4 had a similar safety profile as the licensed IIV3 and that including a second B strain lineage in IIV4 provided superior immunogenicity for the added B strain without affecting the immunogenicity of the three IIV3 strains.

## Introduction

Current trivalent influenza vaccines contain a single B strain, but since the 1980s, two distinct genetic lineages of influenza B virus, Victoria and Yamagata, have been co-circulating worldwide, both of which are responsible for influenza illnesses.^1,2^ Every year, based on surveillance data, the World Health Organization recommends the A and B strains to be included in the next season's influenza vaccines, but selecting the correct B strain has been difficult, resulting in frequent mismatches between the trivalent vaccine and the predominant circulating B-strain lineage. For example, in the US, in half of the Northern Hemisphere influenza seasons between 1999–2000 and 2011–2012, the B-strain lineage included in the trivalent vaccine was not the same as the predominant circulating B lineage.^3^ Similarly, B-strain lineage mismatches occurred in Canada in seven out of the 12 influenza seasons between 2001–2002 and 2012–2013^4^ and in five of the 10 influenza seasons in Europe between 2001–2002 and 2010–2011.^5^

Influenza B disproportionately affects children and older adults, although it can cause illness as severe as influenza A in all age groups.^6-11^ Quadrivalent influenza vaccines containing both B lineages are becoming available and should help address the problem of mismatches between circulating and vaccine B strains.^5^ A quadrivalent split-virion inactivated influenza vaccine (IIV4; VaxigripTetra™, Sanofi Pasteur, Lyon, France) obtained marketing approval in Europe in June 2016 for individuals 36 months of age and older. Phase III clinical trials in younger adults (18–60 years), older adults (>60 years), and children 3 to 8 years of age have shown that IIV4 was as immunogenic as the comparator trivalent inactivated influenza vaccine (IIV3) for each of the three shared influenza strains and superior for the additional B strain.^12-14^ In addition, IIV4 had a safety profile similar to that of the licensed IIV3.^12-14^ A recent systematic review and meta-analysis, which included the results of five randomized clinical trials performed in adults comparing IIV4 to IIV3, came to the same conclusions.^15^ Thus, the addition of the second B-strain lineage to IIV3 is expected to provide added protection against influenza without affecting protection against the original three strains.

Here, we present the results of a study designed to confirm these observations in younger and older adults and to demonstrate lot-to-lot consistency of three commercial batches of the 2014–2015 Northern Hemisphere formulation of IIV4. We also describe antibody persistence up to one year post-vaccination and how vaccination the previous year and high-risk conditions affect the vaccine's immunogenicity.

## Results

### Participants

#### Disposition

**Figure 1.**
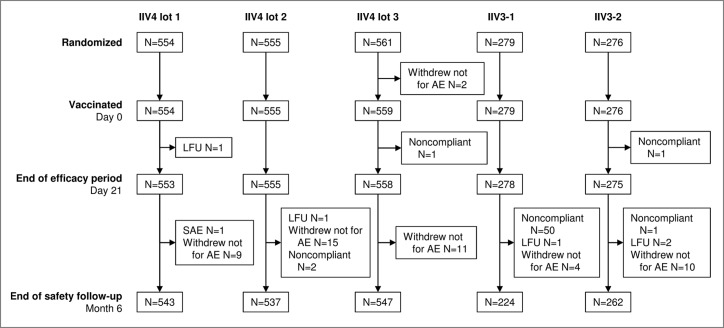
Disposition of participants in the study. 2225 participants were included and randomized 2:2:2:1:1 to receive a single dose of one of the three lots of the 2014–2015 formulation of IIV4, IIV3-1, or IIV3-2. IIV4 contained the A(H1N1), A(H3N2), B Victoria lineage, and B Yamagata lineage strains; IIV3-1 contained the two A strains and the B Victoria lineage strain (IIV3-1); IIV3-2 was the 2014–2015 licensed IIV3 and contained the two A strains and the B Yamagata lineage strain. All but three participants were vaccinated. Reasons for discontinuation included a severe adverse event (SAE), voluntary withdrawal not for an adverse event (AE), noncompliance with the study procedures, and loss to follow-up (LFU). The high number of protocol violations in the IIV3-1 group after day 21 and before month 6 was due to 47 participants who were offered and accepted to receive a second vaccination with the commercial vaccine (IIV3-2) so that they would be covered for the B/Yamagata-lineage strain.

A total of 2225 participants were included between September 17 and October 21, 2014 at three centers in Belgium (n = 468), three in France (n = 560), four in Germany (n = 589), and five in Poland (n = 608) between September 2014 and October 2015 (Fig. 1). This included approximately equal numbers of younger adults (18–60 y, n = 1114) and older adults (>60 y, n = 1111). A total of 2113 participants completed up to month 12, and the study ended on October 23, 2015. The main reason for early discontinuation was voluntary withdrawal unrelated to an adverse event (AE).

#### Baseline characteristics

Mean ages, ethnicity, and proportions of males and females were similar in all study groups (Table 1). Also, in each of the previous three influenza seasons (2013–2014, 2012–2013, and 2011–2012), roughly one-third of participants in each group had received the seasonal influenza vaccine and 0.5% to 1.5% had been diagnosed with influenza. Although vaccination rates were balanced between the study groups, the rates were consistently lower in younger participants (approx. 20% to 25%) than in older participants (approx. 50%) (data not shown).

**Table 1.** Participant baseline characteristics.

Characteristic	IIV4 lot 1	IIV4 lot 2	IIV4 lot 3	IIV3-1	IIV3-2
Randomized, N					
Total	554	555	561	279	276
Younger adults (18–60 y)	278	277	281	140	138
Older adults (>60 y)	276	278	280	139	138
Age (y), mean ± standard deviation	54.5 ± 18.1	54.8 ± 18.2	54.5 ± 18.1	55.6 ± 16.9	53.7 ±18.6
Sex, n (%)					
Male	244 (44.1)	251 (45.2)	276 (49.5)	126 (45.3)	124 (45.1)
Female	309 (55.9)	304 (54.8)	282 (50.5)	152 (54.7)	151 (54.9)
Ethnicity, n(%)					
White/Caucasian	551 (99.6)	548 (98.7)	555 (99.5)	275 (98.9)	271 (98.5)
Other	2 (0.4)	7 (1.3)	3 (0.5)	3 (1.1)	4 (1.5)
History of influenza vaccination, n (%)					
2013–2014	187 (33.8)	196 (35.3)	195 (34.9)	102 (36.7)	89 (32.4)
2012–2013	177 (32.0)	199 (35.9)	199 (35.7)	105 (37.8)	97 (35.3)
2011–2012	179 (32.4)	209 (37.7)	197 (35.3)	104 (37.4)	94 (34.2)
History of influenza diagnosis, n (%)					
2013–2014	3 (0.5)	3 (0.5)	7 (1.3)	3 (1.1)	3 (1.1)
2012–2013	7 (1.3)	5 (0.9)	8 (1.4)	4 (1.4)	3 (1.1)
2011–2012	7 (1.3)	6 (1.1)	3 (0.5)	4 (1.4)	4 (1.5)

Values are for all participants vaccinated. Abbreviations: IIV3-1, the trivalent inactivated influenza vaccine containing the B Victoria lineage strain; IIV3-2, the 2014–2015 licensed IIV3 containing the B Yamagata lineage strain; IIV4, the 2014–2015 formulation of the quadrivalent inactivated influenza vaccine.

### Hemagglutination inhibition (HAI) antibody response to vaccination with IIV4

**Figure 2.**
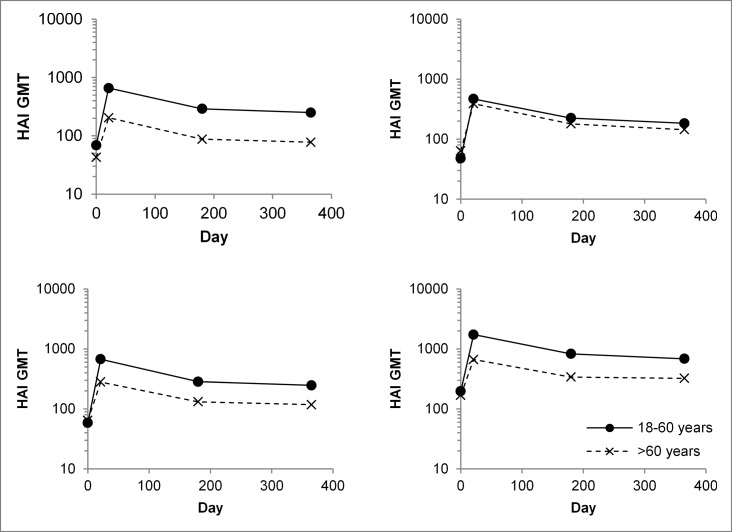
Persistence of hemagglutination inhibition (HAI) antibody titers up to 1 year after vaccination with the quadrivalent inactivated influenza vaccine (IIV4). HAI geometric mean antibody titers (GMTs) for younger adult (18–60 years) and older adult (>60 years) participants vaccinated with IIV4 are shown at baseline (day 0), the end of the primary efficacy assessment period (day 21), the end of the safety follow-up period (month 6), and month 12. Values are for all subjects vaccinated and with data available.

When assessed 21 days after vaccination, IIV4 increased HAI antibody titers for all vaccine strains by 7- to 12-fold in younger adults and by 4- to 6-fold in older adults (Table 2). Seroprotection rates on day 21 were at least 98% in younger adults and at least 90% in older adults. Rates of seroconversion/significant increase in titer were 64% to 71% in younger adults and 43% to 48% in older adults. After day 21, HAI antibody titers gradually decreased so that they were 2- to 3-fold lower at 1 year than at day 21 (Fig. 2), although they remained above baseline, with seroprotection rates for all vaccine strains above 90% in younger adults and above 75% in older adults

**Table 2.** HAI antibody responses.

			A/H1N1		A/H3N2		B Victoria lineage		B Yamagata lineage	
Age	Measure	Day	Pooled IIV4	Pooled IIV3	Pooled IIV4	Pooled IIV3	Pooled IIV4	IIV3-1	Pooled IIV4	IIV3-2
18–60 y	N	—	833	278	832	278	832	140	832	138
	GMT (95% CI)	0	62.2 (55.6; 69.7)	66.7 (54.9; 80.9)	48.6 (43.3; 54.4)	42.2 (34.8; 51.0)	61.3 (55.3; 67.8)	64.5 (50.1; 83.0)	233 (210; 259)	285 (222; 365)
		21	608 (563; 657)	685 (587; 800)	498 (459; 541)	629 (543; 728)	708 (661; 760)	735 (615; 879)	1715 (1607; 1830)	1735 (1490; 2019)
	Seroprotection^a^, % (95% CI)	0	64.6 (61.2; 67.8)	68.3 (62.5; 73.8)	58.5 (55.1; 61.9)	55.8 (49.7; 61.7)	61.9 (58.5; 65.2)	67.9 (59.4; 75.5)	88.0 (85.6; 90.1)	90.6 (84.4; 94.9)
		21	98.2 (97.0; 99.0)	97.1 (94.4; 98.7)	98.0 (96.7; 98.8)	98.6 (96.4; 99.6)	99.8 (99.1; 100.0)	100.0 (97.4; 100.0)	100.0 (99.6; 100.0)	100.0 (97.4; 100.0)
	GMTR (95% CI)	21/0	9.77 (8.69; 11.0)	10.3 (8.35; 12.7)	10.3 (9.15; 11.5)	14.9 (12.1; 18.4)	11.6 (10.4; 12.9)	11.4 (8.66; 15.0)	7.35 (6.66; 8.12)	6.08 (4.79; 7.72)
	Seroconversion or significant increase, % (95% CI)	21/0	64.1 (60.7; 67.4)	65.1 (59.2; 70.7)	66.2 (62.9; 69.4)	73.4 (67.8; 78.5)	70.9 (67.7; 74.0)	70.0 (61.7; 77.4)	63.7 (60.3; 67.0)	60.9 (52.2; 69.1)
>60 y	N	—	832	275	831	274	831	138	831	137
	GMT (95% CI)	0	44.3 (39.9; 49.2)	44.4 (36.7; 53.6)	64.1 (57.3; 71.7)	70.8 (57.8; 86.7)	62.2 (56.2; 68.9)	65.4 (51.1; 83.7)	159 (145; 175)	170 (132; 217)
		21	219 (199; 241)	268 (228; 314)	359 (329; 391)	410 (352; 476)	287 (265; 311)	301 (244; 372)	655 (611; 701)	697 (593; 820)
	Seroprotection^a^, % (95% CI)	0	57.7 (54.3; 61.1)	59.3 (53.2; 65.1)	65.8 (62.5; 69.0)	67.9 (62.0; 73.4)	65.9 (62.6; 69.2)	70.3 (61.9; 77.8)	86.3 (83.8; 88.5)	86.9 (80.0; 92.0)
		21	90.6 (88.4; 92.5)	94.5 (91.2; 96.9)	96.1 (94.6; 97.4)	97.8 (95.3; 99.2)	96.5 (95.0; 97.7)	95.7 (90.8; 98.4)	100.0 (99.6; 100.0)	100.0 (97.3; 100.0)
	GMTR (95% CI)	21/0	4.94 (4.46; 5.47)	6.03 (4.93; 7.37)	5.60 (5.02; 6.24)	5.79 (4.74; 7.06)	4.61 (4.18; 5.09)	4.60 (3.50; 6.05)	4.11 (3.73; 4.52)	4.11 (3.19; 5.30)
	Seroconversion or significant increase, % (95% CI)	21/0	45.6 (42.1; 49.0)	50.2 (44.1; 56.2)	47.5 (44.1; 51.0)	48.5 (42.5; 54.6)	45.2 (41.8; 48.7)	43.5 (35.1; 52.2)	42.7 (39.3; 46.2)	38.7 (30.5; 47.4)

Values are for all participants vaccinated with available pre- and post-vaccination HAI titers. Abbreviations: CI, confidence interval; GMT, geometric mean titer; GMTR, geometric mean of the individual ratios of the post-vaccination (day 21) HAI titer divided by the pre-vaccination (day 0) HAI titer; HAI, hemagglutination inhibition; IIV3, trivalent inactivated influenza vaccine; IIV4, quadrivalent inactivated influenza vaccine.

a Seroprotection was defined as a HAI titer ≥40.

b Seroconversion was defined as a pre-vaccination (day 0) HAI titer <10 and a post-vaccination (day 21) HAI titer ≥1:40, and a significant increase was defined as a pre-vaccination HAI titer ≥10 and a ≥4-fold increase in HAI titer.

#### Non-inferiority and superiority of IIV4 vs. IIV3 and lot-to-lot equivalence

Post-vaccination HAI geometric mean titers (GMTs) for all four vaccine strains were statistically equivalent between the three IIV4 lots (Table 3). In both age groups, post-vaccination (day 21) HAI GMTs for the pooled IIV4 lots were statistically non-inferior to those for the IIV3 for the A strains and for the B strain when it was included in the comparator IIV3 (Table 4). Furthermore, for the additional B lineage strain, HAI antibody titers induced by IIV4 were statistically superior to those induced by the comparator IIV3 lacking it (Table 3).

**Table 3.** Non-inferiority and superiority of post-vaccination (day 21) HAI antibody responses for pooled quadrivalent vs. trivalent vaccines.

Comparison	Strain	Comparator	Age group	Ratio of day 21 HAI GMTs	Non-inferior^a^	Superior^b^
Non-inferiority of IIV4 vs. IIV3	A(H1N1)	Pooled IIV3s	18–60 y	0.894 (0.762, 1.05)		
			>60 y	0.817 (0.675, 0.990)		
			Overall	0.855 (0.754; 0.968)	Yes	—
	A(H3N2)	Pooled IIV3s	18–60 y	0.797 (0.675, 0.940)		
			>60 y	0.876 (0.737. 1.04)		
			Overall	0.835 (0.741; 0.941)	Yes	—
	B Victoria lineage	IIV3-1	18–60 y	0.963 (0.800, 1.16)		
			>60 y	0.954 (0.768, 1.19)		
			Overall	0.959 (0.831; 1.11)	Yes	—
	B Yamagata lineage	IIV3-2	18–60 y	0.987 (0.832, 1.17)		
			>60 y	0.940 (0.784, 1.13)		
			Overall	0.964 (0.850; 1.09)	Yes	—
Superiority of IIV4 vs. IIV3	B Victoria lineage	IIV3-2	18–60 y	3.48 (2.89; 4.19)	—	Yes
			>60 y	2.36 (1.91; 2.93)	—	Yes
	B Yamagata lineage	IIV3-1	18–60 y	2.49 (2.08; 2.98)	—	Yes
			>60 y	1.86 (1.55; 2.24)	—	Yes

Abbreviations: CI, confidence interval; GMT, geometric mean titer; HAI, hemagglutination inhibition; IIV3-1, trivalent inactivated influenza vaccine containing the B Victoria lineage strain; IIV3-2, licensed IIV3 containing the B Yamagata lineage strain; IIV4, quadrivalent inactivated influenza vaccine.

a Non-inferiority was assessed in participants completing the study according to protocol and was concluded if the lower limit of the overall age-stratified two-sided 95% CI of the ratio of post-vaccination (day 21) GMTs between groups (pooled IIV4 lots/IIV3 comparator(s)) was >0.667 for each strain.

b Superiority was assessed in all vaccinated participants and was concluded if the lower limit of the two-sided 95% CI of the ratio of post-vaccination (day 21) GMTs between groups (pooled IIV4 lots/IIV3 comparator) was >1 for each B strain and in each age group.

**Table 4.** IIV4 lot equivalence.

Strain	Age group	Lot	N	HAI GMT (95% CI)	Age-stratified ratio of day 21 HAI GMTs (95% CI)	Equivalent^a^
A(H1N1)	18–60 y	1	275	609 (529; 699)		
		2	275	654 (572; 748)		
		3	278	578 (509; 655)		
	>60 y	1	276	222 (186; 264)		
		2	276	215 (183; 253)		
		3	278	219 (185; 259)		
	All	1 vs. 2			0.979 (0.840; 1.14)	Yes
		1 vs. 3			1.03 (0.887; 1.20)	Yes
		2 vs. 3			1.06 (0.910; 1.22)	Yes
A(H3N2)	18–60 y	1	275	529 (457; 613)		
		2	274	489 (423; 565)		
		3	278	486 (423; 559)		
	>60 y	1	276	362 (312; 420)		
		2	275	394 (341; 456)		
		3	278	324 (278; 377)		
	Overall	1 vs. 2			0.996 (0.861; 1.15)	Yes
		1 vs. 3			1.10 (0.953; 1.28)	Yes
		2 vs. 3			1.11 (0.958; 1.28)	Yes
B Victoria lineage	18–60 y	1	275	712 (632; 803)		
		2	274	722 (638; 816)		
		3	278	691 (610; 782)		
	>60 y	1	276	272 (236; 314)		
		2	276	314 (273; 360)		
		3	277	278 (241; 321)		
	Overall	1 vs. 2			0.926 (0.812; 1.06)	Yes
		1 vs. 3			1.01 (0.881; 1.15)	Yes
		2 vs. 3			1.09 (0.952; 1.24)	Yes
B Yamagata lineage	18–60 y	1	275	1734 (1550; 1940)		
		2	274	1738 (1552; 1948)		
		3	278	1667 (1485; 1872)		
	>60 y	1	276	595 (527; 672)		
		2	275	662 (588; 745)		
		3	278	715 (634; 806)		
	Overall	1 vs. 2			0.947 (0.843; 1.06)	Yes
		1 vs. 3			0.93 (0.827; 1.05)	Yes
		2 vs. 3			0.983 (0.874; 1.10)	Yes

Values are for all participants completing the study according to protocol. Abbreviations: CI, confidence interval; GMT, geometric mean titer; GMTR, geometric mean of the individual ratios of the post-vaccination (day 21) HAI titer divided by the pre-vaccination (day 0) HAI titer; HAI, hemagglutination inhibition

a Equivalence was assessed in all participants vaccinated and was concluded if the limits of the overall age-stratified two-sided 95% CI of the ratio of the GMTs between batches were between the interval from 0.667 and 1.5.

#### Influence of previous vaccination on HAI antibody titers

As expected, baseline HAI GMTs were higher in participants who had received the previous season's influenza vaccine than in those who had not (Table 5). IIV4 induced post-vaccination (day 21) GMTs for each vaccine strain that were 1.3- to 2.2-fold higher in participants who had not received the previous season's influenza vaccine than in those who had received it. Regardless, for participants who received the previous year's vaccination, IIV4 induced post-vaccination seroprotection rates against all strains that remained above 98% for younger adults and above 91% for older adults (Table 6).

**Table 5.** HAI GMTs at baseline and day 21 by previous year's vaccination.

			HAI GMT (95% CI)							
			A/H1N1		A/H3N2		B Victoria lineage		B Yamagata lineage	
Age	Vaccine	Day	Vaccinated previous year	Not vaccinated previous year	Vaccinated previous year	Not vaccinated previous year	Vaccinated previous year	Not vaccinated previous year	Vaccinated previous year	Not vaccinated previous year
18–60 y	Pooled IIV4		N = 173	N = 659	N = 173	N = 658	N = 173	N = 658	N = 173	N = 658
		0	169 (138, 206)	47.8 (42.2, 54.2)	160 (130, 198)	35.5 (31.4, 40.1)	156 (129, 189)	47.8 (42.8, 53.5)	532 (448, 633)	187 (167, 210)
		21	355 (305, 413)	701 (644, 764)	395 (338, 461)	530 (482, 583)	495 (424, 578)	779 (721, 842)	1077 (933, 1244)	1938 (1806, 2080)
	Pooled IIV3		N = 60	N = 218	N = 60	N = 218	N = 36	N = 104	N = 24	N = 114
		0	160 (113, 228)	52.4 (42.2, 65.2)	147 (104, 208)	29.9 (24.4, 36.6)	171 (112, 261)	46.0 (34.7, 61.0)	570 (344, 944)	246 (187, 325)
		21	365 (274, 488)	815 (684, 971)	387 (282, 532)	719 (611, 846)	484 (346, 677)	850 (692, 1044)	973 (657, 1441)	1959 (1673, 2294)
>60 y	Pooled IIV4		N = 404	N = 424	N = 404	N = 423	N = 403	N = 424	N = 404	N = 423
		0	76.9 (67.4, 87.7)	26.0 (22.5, 30.0)	113 (99.2, 129)	36.9 (31.3, 43.5)	100 (89.2, 113)	39.2 (33.7, 45.6)	274 (248, 304)	94.5 (82.1, 109)
		21	176 (156, 197)	270 (232, 314)	275 (247, 306)	462 (406, 525)	221 (198, 246)	367 (327, 412)	509 (464, 558)	835 (758, 921)
	Pooled IIV3		N = 131	N = 143	N = 131	N = 142	N = 66	N = 71	N = 65	N = 72
		0	86.4 (67.9, 110)	24.2 (18.8, 31.2)	143 (114, 180)	37.5 (28.0, 50.3)	97.7 (76.0, 125)	45.0 (30.0, 67.5)	300 (232, 389)	101 (69.7, 147)
		21	173 (142, 212)	399 (316, 504)	305 (254, 367)	543 (432, 683)	190 (153, 237)	459 (329, 640)	462 (368, 580)	1011 (830, 1232)

Values are for all participants vaccinated with available pre- and post-vaccination HAI titers. Abbreviations: CI, confidence interval; GMT, geometric mean titer; HAI, hemagglutination inhibition; IIV3, trivalent inactivated influenza vaccine; IIV4, quadrivalent inactivated influenza vaccine.

**Table 6.** Post-vaccination (day 21) seroprotection rates by previous year's vaccination.

		Seroprotection, % (95% CI)							
		A/H1N1		A/H3N2		B Victoria lineage		B Yamagata lineage	
Age	Vaccine	Vaccinated previous year	Not vaccinated previous year	Vaccinated previous year	Not vaccinated previous year	Vaccinated previous year	Not vaccinated previous year	Vaccinated previous year	Not vaccinated previous year
18– 60 y	Pooled IIV4	N = 173	N = 659	N = 173	N = 658	N = 173	N = 658	N = 173	N = 658
		98.3 (95.0, 99.6)	98.2 (96.8, 99.1)	98.8 (95.9, 99.9)	97.7 (96.3, 98.7)	99.4 (96.8, 100.0)	99.8 (99.2, 100.0)	100.0 (97.9, 100.0)	100.0 (99.1, 100.0)
	Pooled IIV3	N = 60	N = 218	N = 60	N = 218	N = 36	N = 104	N = 24	N = 114
		98.3 (91.1, 100.0)	96.8 (93.5, 98.7)	98.3 (91.1, 100.0)	98.6 (96.0, 99.7)	100.0 (96.5, 100.0)	100.0 (96.5, 100.0)	100.0 (96.8, 100.0)	100.0 (96.8, 100.0)
>60 y	Pooled IIV4	N = 404	N = 131	N = 404	N = 131	N = 403	N = 66	N = 404	N = 65
		91.3 (88.2, 93.9)	93.9 (88.3, 97.3)	97.0 (94.9, 98.5)	98.5 (94.6, 99.8)	95.5 (93.0, 97.3)	97.0 (89.5, 99.6)	100.0 (99.1, 100.0)	100.0 (94.5, 100.0)
	Pooled IIV3	N = 424	N = 143	N = 423	N = 142	N = 424	N = 71	N = 423	N = 71
		89.9 (86.6, 92.6)	95.1 (90.2, 98.0)	95.3 (92.8, 97.1)	97.2 (92.9, 99.2)	97.4 (95.4, 98.7)	94.4 (86.2, 98.4)	100.0 (99.1, 100.0)	94.4 (86.2, 98.4)

Values are for all participants vaccinated with available pre- and post-vaccination HAI titers. Seroprotection was defined as a hemagglutination inhibition titer ≥40. Abbreviations: CI, confidence interval; IIV3, trivalent inactivated influenza vaccine; IIV4, quadrivalent inactivated influenza vaccine.

#### Influence of high-risk conditions on HAI antibody titers

In both age groups, baseline and post-vaccination GMTs induced by IIV4 were unaffected by the presence of high-risk conditions (Table S1).

### Seroneutralizing (SN) antibody response to vaccination with IIV4

The SN antibody response was examined in a randomly selected subset of participants as part of an exploratory analysis. Vaccination with IIV4 increased SN antibody titers for all strains, with a ≥4-fold increase in SN antibody titers against each vaccine strain achieved in 47%–70% of younger adults and 33%–55% of older adults (Table 7).

**Table 7.** SN antibody responses.

			IIV4				IIV3			
Age	Measure	Day	A/H1N1	A/H3N2	B Victoria lineage	B Yamagata lineage	A/H1N1	A/H3N2	B Victoria lineage	B Yamagata lineage
18–60 y	N	—	150	150	150	150	100	100	50	50
	GMT (95% CI)	0	265 (188, 374)	46.7 (39.1, 55.8)	96.3 (75.3, 123)	143 (107, 190)	238 (159, 356)	43.8 (36.5, 52.5)	111 (71.2, 174)	188 (125, 281)
		21	3540 (2997, 4183)	215 (182, 254)	1143 (952, 1373)	1825 (1463, 2277)	3076 (2308, 4100)	307 (239, 395)	1269 (875, 1841)	1680 (1164, 2423)
	GMTR (95% CI)	21/0	13.4 (9.61, 18.6)	4.60 (3.81, 5.56)	11.9 (9.24, 15.2)	12.8 (9.64, 17.0)	12.9 (8.89, 18.8)	7.01 (5.29, 9.30)	11.4 (7.08, 18.3)	8.95 (5.96, 13.4)
	≥4-fold rise, n (%)	21/0	61.3 (53.0, 69.2)	47.3 (39.1, 55.6)	70.0 (62.0, 77.2)	67.3 (59.2, 74.8)	62.0 (51.7, 71.5)	59.0 (48.7, 68.7)	66.0 (51.2, 78.8)	68.0 (53.3, 80.5)
≥60 y	N	—	150	150	150	149	98	98	49	49
	GMT (95% CI)	0	137 (101; 187)	48.7 (39.7; 59.7)	114 (88.7; 147)	124 (97.1; 158)	154 (101; 234)	58.5 (44.7; 76.5)	109 (78.2; 151)	136 (85.0; 218)
		21	988 (763; 1279)	179 (151; 212)	509 (414; 625)	572 (465; 704)	1196 (902; 1584)	192 (149; 246)	559 (391; 799)	523 (370; 738)
	GMTR (95% CI)	21/0	7.19 (5.59; 9.24)	3.67 (3.00; 4.50)	4.46 (3.60; 5.53)	4.68 (3.67; 5.96)	7.76 (5.38; 11.2)	3.28 (2.57; 4.17)	5.14 (3.34; 7.93)	3.84 (2.57; 5.74)
	≥4-fold rise, n (%)	21/0	54.7 (46.3; 62.8)	33.3 (25.9; 41.5)	42.7 (34.6; 51.0)	41.6 (33.6; 50.0)	52.0 (41.7; 62.2)	34.7 (25.4; 45.0)	36.7 (23.4; 51.7)	34.7 (21.7; 49.6)

Values are for all participants with pre- and post-vaccination seroneutralization titers available. Abbreviations: CI, confidence interval; GMT, geometric mean SN titer; GMTR, geometric mean of the individual ratios of the post-vaccination (day 21) SN titer divided by the pre-vaccination (day 0) SN titer; IIV3, trivalent inactivated influenza vaccine; IIV4, quadrivalent inactivated influenza vaccine; NC, not calculable; SN, seroneutralizing.

#### Safety and tolerability

#### Solicited reactions

Solicited reactions were mostly grade 1 and were most commonly injection-site pain, headache, malaise, and myalgia (Supplemental Table S2). Most of these reactions resolved within 3 days (data not shown). Within each age group, proportions with solicited reactions or grade 3 solicited reactions were similar between IIV3 and IIV4. Rates of solicited reactions were lower in older adults than in younger adults for both IIV3 and IIV4.

#### Unsolicited AEs

Rates of unsolicited AEs and serious adverse events (SAEs) were similar between the different vaccination groups (Supplemental Table S3). No SAEs considered related to vaccination were reported up to the end of the safety follow-up at month 6. One participant left the study before the end of the safety follow-up period due to an unrelated SAE. A single adverse event of special interest (AESI), convulsion 13 days after vaccination resulting in hospitalization that resolved the same day, was reported for a participant vaccinated with IIV4. The participant had a history of convulsive seizure before enrollment in the study, and the event was not considered to be related to vaccination.

## Discussion

This study confirmed that for younger and older adults, adding a second B strain lineage to IIV3 to produce IIV4 provides a superior response to the added B strain lineage without affecting the antibody response induced by the original three vaccine strains. Despite relatively high seroprotection rates at baseline, vaccination with IIV4 increased GMTs from baseline for all four vaccine strains by at least 4-fold in older adults and by at least 7-fold in younger adults. HAI antibody titers were highest 21 days after vaccination but remained above baseline for at least 1 year, in agreement with previous studies of split-virion IIV3s in healthy adults^16^ and pregnant women.^17^

As found in previous studies and concluded in a recent meta-analysis of clinical trials.^15,12,13^, this study showed that IIV4 is well tolerated and has a similar safety profile to IIV3. Importantly, IIV4 did not cause any vaccine-related SAEs or generate any new safety signals. As found in other studies of inactivated influenza vaccines,^18,19^ reactogenicity was lower in older adults than in younger adults. Consistent with other studies, antibody responses were also lower in older adults.^19,20^

The current study, performed in France, Belgium, Germany, and Poland, examined the 2014–2015 Northern Hemisphere formulation of IIV4. Very similar results were found in a study of younger adults vaccinated with the 2011–2012 formulation in France and Germany.^12^ As in the current study, the previous study demonstrated non-inferior HAI antibody responses for IIV4 compared to the licensed IIV3 for all matched strains and superior antibody responses for B lineages when compared to an IIV3 containing the alternate B strain lineage. Also, as in the current study, a study performed in Australia and the Philippines found robust, equivalent antibody responses to three lots of the 2011–2012 Northern Hemisphere formulation of IIV4 in children, adolescents, and younger adults.^13^ This suggests that IIV4s can be produced consistently across seasons and manufacturers.

Rates of serious influenza illness are known to be higher and vaccine immune responses lower in older adults because of immunosenescence, the age-related decline of the immune system.^21^ This higher rate of serious influenza illness in older adults is probably also due to the higher incidence of certain conditions that increase the risk of complications.^22,23^ In the current study, however, conditions that increase the risk of complications did not result in lower immune responses to the vaccines.

This study showed that HAI antibody titers to each strain were lower in participants vaccinated with the previous season's influenza vaccine. This was also reported in another study of children 3 to 8 years of age vaccinated with the 2013–2014 Northern Hemisphere formulations of IIV3 and IIV4.^14^ The clinical significance and reason for this finding is not clear, but for participants in the current study who had received the previous year's vaccination, post-vaccination seroprotection rates against all strains remained above 98% for younger adults and above 90% for older adults.

Seroprotection is commonly defined as an HAI antibody titer of ≥40.^24,25^ According to this definition, seroprotection against all strains was attained by at least 90% of younger and older adults in this study. Some authors have suggested, however, that serologic results overestimate protection by IIVs, and they argue that fixed HAI titer cut-offs, at least alone, are not appropriate for estimating protection.^26-30^ Because of these limitations, in Europe, SN responses must now be reported in addition to HAI antibody responses.^31^ SN responses are strongly associated with protection, and SN titers correlate well with HAI antibody titers, although the combination of SN and HAI is better than either alone at describing vaccine efficacy.^32,33^ Here, we showed that vaccination with IIV4 induced SN antibody responses to each of four vaccine strains of influenza in most of the participants tested, even those that had detectable SN antibodies at baseline. This strengthens the conclusion that, in most participants, IIV4 induced protective antibodies against all four vaccine strains of influenza. Because a correlate of protection based on SN titer has not been established, these results cannot be used to estimate or compare efficacy between vaccines.^28^

Quadrivalent influenza vaccines are gradually replacing trivalent vaccines. The current study confirmed the immunogenicity, safety profile, and lot-to-lot consistency of the 2014–2015 Northern Hemisphere formulation of IIV4. It also showed that the vaccine should provide good protection against all four included strains of influenza, even in individuals with high-risk conditions and individuals vaccinated the previous year for seasonal influenza. By providing broader coverage and a better match to circulating B strains, IIV4 should help reduce the public health impact of influenza.

## Patients and methods

### Study design

This was a phase III randomized clinical trial carried out at three centers in Belgium, three in France, four in Germany, and five in Poland between September 2014 and October 2015 (EudraCT no. 2014-000785-21). Primary objectives were (1) to demonstrate equivalence of the post-vaccination (day 21) HAI antibody response induced by the three different lots of IIV4 for each vaccine strain; and (2) to demonstrate non-inferiority of the post-vaccination (day 21) HAI antibody response induced by the pooled IIV4 lots compared with the IIV3 containing the Victoria lineage strain (IIV3-1) and the IIV3 containing the Yamagata lineage strain (IIV3-2). Secondary objectives were to confirm superiority of the HAI antibody response to the B Victoria lineage strain in the IIV4 group compared to the response to the B Victoria lineage strain in the IIV3-2 group, 21 days after vaccination in younger and older adult age groups; confirm superiority of the response to the B Yamagata lineage in the IIV4 group compared to the response to the B Yamagata lineage strain in the IIV3-1 group; and to describe the safety profile, including solicited reactions during the 7 days following vaccination, AEs during the 21 days following vaccination, and SAEs and AESIs during the 6 months after vaccination. Antibody persistence up to 12 months after vaccination was investigated as an observational objective.

### Ethics

The study was approved by each institution's ethics committee or review board. The conduct of this trial was consistent with the standards established by the Declaration of Helsinki and complied with the International Conference on Harmonization Guidelines for Good Clinical Practice as well as all local and national regulations and directives. All participants provided written informed consent to be included in this trial.

### Participants

The study included healthy adults (≥18 years). Potential participants were excluded if they received any vaccine in the 4 weeks preceding trial vaccination or planned to receive any vaccine in the 3 weeks following trial vaccination; had previously received the 2014 Southern Hemisphere formulation or the 2014–2015 Northern Hemisphere formulation of the seasonal influenza vaccine; received immune globulins, blood, or blood-derived products in the past 3 months; had known or suspected congenital or acquired immunodeficiency; received immunosuppressive therapy within the preceding 6 months; received long-term systemic corticosteroid therapy (prednisone or equivalent for >2 consecutive weeks) within the past 3 months; had known systemic hypersensitivity to any of the vaccine components or a previous life-threatening reaction to the vaccines used in the trial or to a vaccine containing any of the same substances; had thrombocytopenia; had a bleeding disorder or received anticoagulants in the 3 weeks before the study; had a chronic illness that, in the opinion of the investigator, might interfere with the study assessments; or had moderate or severe acute illness or infection on the day of vaccination or febrile illness (temperature ≥38.0°C). Women were excluded if they were pregnant, lactating, or of childbearing potential and not using an effective method of birth control. Prior to enrollment, all participants were assessed for preexisting conditions and illnesses. Participants were considered to be at risk for influenza-related complications if they had at least one past or current high-risk condition as defined by the US Centers for Disease Control and Prevention.^34^

### Vaccines

As recommended by the World Health Organization and the European Union for the 2014–2015 Northern Hemisphere influenza season, IIV4 (VaxigripTetra, Sanofi Pasteur, Lyon, France) included A/California/7/2009 (H1N1)pdm09, A/Texas/50/2012 (H3N2), B/Brisbane/60/2008 (B Victoria lineage), and B/Massachusetts/2/2012 (B Yamagata lineage). IIV3-1 contained the two above A strains and the B Victoria lineage strain. IIV3-2 was the 2014–2015 licensed IIV3 (Vaxigrip, Sanofi Pasteur, Lyon, France) and contained the two above A strains and the B Yamagata lineage strain. All vaccines were thimerosal-free, inactivated, split-virion, and each 0.5-mL dose of vaccine contained 15 μg of hemagglutinin from each strain.

### Vaccination

Participants were randomized 2:2:2:1:1 to receive a single dose of one of the three lots of IIV4, IIV3-1, or IIV3-2. The randomization list was generated by computer using the permuted block method with stratification by site and age group. Participants were assigned via an interactive voice or web response system. The study was double-blinded (investigator and participants) for the IIV4 or IIV3-2 groups. The IIV3-1 group was single-blinded because it was delivered in different packaging than the investigational products, although it was presented to participants in an identical 0.5-mL single-use syringe. For all participants, immunogenicity was assessed in a blinded manner.

### HAI assay

The primary immunogenicity endpoint was the HAI titer measured 21 days after vaccination. HAI titers were also assessed at baseline (day 0), at the end of the safety follow-up period (month 6), and at month 12. HAI titers were measured as described previously.^12^ Hemagglutinin antigens used in the HAI assay corresponded to the strains in IIV4. Briefly, the highest serum dilution resulting in complete inhibition of hemagglutination was determined for duplicates of each sample. The HAI antibody titer for each sample was calculated as the geometric mean of the reciprocal of the duplicate values. The lower limit of quantitation was set at the reciprocal of the lowest dilution used in the assay (10), and the upper limit of quantitation as the highest dilution used in the assay (10,240). GMTs, geometric means of individual titer ratio of post-vaccination vs. pre-vaccination (day 0), seroprotection, and seroconversion or significant increase in HAI titer were calculated. Seroprotection was defined as a HAI titer ≥40; seroconversion as a HAI titer <10 on day 0 and a post-vaccination HAI titer ≥ 40; and significant increase was defined as a HAI titer ≥ 10 on day 0 and a ≥ 4-fold post-vaccination increase in HAI titer.

### SN assay

SN titers were measured using the World Health Organization procedure^35^ in 50 randomly selected participants in each age and vaccine group. Briefly, serially diluted, heat-inactivated human serum samples were mixed with a fixed amount of challenge virus prior to the addition of Madin-Darby canine kidney cells. Challenge virus strains were the same as those in IIV4. After overnight incubation, the viral nucleoprotein production in infected cells was measured by enzyme linked immunosorbent assay using a monoclonal antibody specific to influenza A or influenza B nucleoprotein. The lower limit of detection was the reciprocal of the lowest dilution used in the assay (1:10), and the upper limit of detection was the reciprocal of the highest dilution used in the assay (1:10,240).

### Safety and reactogenicity

Unsolicited AEs and SAEs were collected according to International Conference on Harmonization E2A Guideline for Clinical Safety Data Management: Definitions and Standards for Expedited Reporting. Safety endpoints included immediate unsolicited AEs (within 30 min after vaccination); solicited injection-site reactions (pain, erythema, swelling, induration, ecchymosis) and systemic reactions (fever, headache, malaise, myalgia, shivering) within 7 days, recorded by patients on diary cards; unsolicited AEs within 21 days; and SAEs and AESIs up to 6 months. Erythema, swelling, induration, and ecchymosis were considered grade 1 for ≥25 to ≤50 mm, grade 2 for ≥51 to ≤100 mm, and grade 3 for >100 mm. Fever was considered grade 1 for ≥38.0°C to ≤38.4°C, grade 2 for ≥38.5°C to ≤38.9°C, and grade 3 for ≥39.0°C. All other reactions and AEs were considered grade 1 for not interfering with activity, grade 2 for some interference with activity, and grade 3 for significant, preventing daily activity. Investigators categorized the relatedness of unsolicited AEs, SAEs, and AESIs as “unrelated” or as “possibly or probably related”. AESIs included anaphylaxis, Guillain-Barré syndrome, encephalitis/myelitis, neuritis, febrile and non-febrile convulsions, thrombocytopenia, and vasculitis.

### Estimation of study size

For each age group, approximately 278 participants were to be enrolled in each IIV4 lot group and 139 participants in each IIV3 group (2224 participants in total). This produced an overall power (i.e., product of all individual power estimates) > 90% to demonstrate the following: (1) equivalence of the three IIV4 lots in terms of GMTs with a one-sided alpha level of 2.5% and an equivalence margin of 1.5, assuming a standard deviation of log_10_-transformed titers of 0.6 for each strain and 90% participants evaluable; (2) non-inferiority of pooled IIV4 lots vs. IIV3 group(s) in terms of GMTs with a one-sided alpha level of 2.5% and a non-inferiority margin of 1.5, assuming a standard deviation of log_10_-transformed titers of 0.6 for each strain and 90% participants evaluable; and (3) superiority in each age group of IIV4 vs. each IIV3 group for the B strain it did not contain assuming that the IIV4 induces at least a 2-fold increase in the IIV3 response to the B strain it did not contain, a standard deviation of log_10_-transformed titers of 0.6, and 95% participants evaluable.

### Statistical analysis

Statistical analysis was performed using SAS® version 9.2 or later (SAS Institute, Cary, NC, USA). Missing or incomplete data were not replaced, with the exception that all HAI titers under the lower limit of quantitation (10) were assigned a value of 5 and all HAI titers above the upper limit of quantitation (10,240) were assigned a value of 10,240. To calculate GMTs, the means and 95% confidence intervals (CIs) were determined from log_10_-transformed data using Student's *t*-distribution with n−1 degrees of freedom, after which antilog transformations were applied to the results of calculations. Lot-to-lot consistency and non-inferiority of IIV4 vs. IIV3 were assessed in all participants who completed the study according to protocol. Superiority of IIV4 vs. IIV3 was assessed primarily in all vaccinated participants. Immunogenicity measures were reported for all randomized participants who received the study vaccine with available pre- and post-vaccination HAI titers. As previously described,^13^ lot-to-lot equivalence for each strain was demonstrated if the age group-stratified two-sided 95% CI of the post-vaccination HAI GMT ratio was between 0.667 and 1.5. Non-inferiority and superiority were assessed as previously described.^12^ Briefly, non-inferiority and superiority assessments were based on the two-sided 95% CI of the ratio of post-vaccination GMTs between the IIV4 and IIV3 group(s). For non-inferiority, the age-stratified CI was calculated using an analysis of variance model of log_10_-transformed titers, with the age group (18–60 and >60 years) as the stratifying factor in the model. Non-inferiority was demonstrated if the lower limit of the age-stratified two-sided 95% CI of the ratio of day 21 GMTs was >0.667 for each strain. For superiority, the CI was calculated using a normal approximation and was demonstrated if the lower limit of the two-sided 95% CI of the ratio of day 21 GMTs was >1 for each B strain in each age group. Safety is presented using descriptive statistics and assessed in all participants who received a study vaccine according to the vaccine actually received.

## Supplementary Material

Supplemental Material
