# Supplementary material for: Safety, immunogenicity, and lot-to-lot consistency of a split-virion quadrivalent influenza vaccine in younger and older adults: A phase III randomized, double-blind clinical trial
Source: Hum Vaccin Immunother. 2017 Nov 27;14(3):596–608. doi: 10.1080/21645515.2017.1384106 (PMC5861783; doi:10.1080/21645515.2017.1384106)
Supplement: Supplemental Material [file khvi-14-03-1384106-s001.docx]

**Supplemental Table S1. HAI GMTs at baseline and day 21 by risk for influenza-related complications**

|  |  |  | **A/H1N1** | | **A/H3N2** | | **B Victoria lineage** | | **B Yamagata lineage** | |
| --- | --- | --- | --- | --- | --- | --- | --- | --- | --- | --- |
| **Age** | **Vaccine** | **Day** | **At risk^a^** | **Not at risk** | **At risk^a^** | **Not at risk** | **At risk^a^** | **Not at risk** | **At risk^a^** | **Not at risk** |
| 18–60 y | Pooled IIV4 |  | N=144 | N=689 | N=144 | N=688 | N=144 | N=688 | N=144 | N=688 |
|  |  | 0 | 58.1 (44.5, 75.9) | 63.2 (55.8, 71.5) | 51.4 (39.2, 67.3) | 48.0 (42.3, 54.5) | 74.6 (57.7, 96.4) | 58.8 (52.6, 65.7) | 189 (148, 241) | 244 (218, 273) |
|  |  | 21 | 534 (435, 656) | 625 (576, 679) | 491 (401, 602) | 500 (457, 547) | 756 (630, 906) | 699 (648, 754) | 1406 (1201, 1646) | 1788 (1664, 1920) |
|  | Pooled IIV3 |  | N=49 | N=229 | N=49 | N=229 | N=27 | N=113 | N=22 | N=116 |
|  |  | 0 | 51.2 (31.8, 82.5) | 70.6 (57.0, 87.3) | 34.5 (21.4, 55.6) | 44.0 (35.7, 54.2) | 55.8 (31.8, 98.1) | 66.8 (50.1, 88.9) | 219 (116, 415) | 300 (228, 394) |
|  |  | 21 | 613 (442, 852) | 702 (589, 837) | 466 (326, 665) | 671 (571, 788) | 817 (511, 1305) | 717 (591, 870) | 1571 (972, 2540) | 1767 (1507, 2073) |
| >60 y | Pooled IIV4 |  | N=454 | N=378 | N=454 | N=377 | N=453 | N=378 | N=453 | N=378 |
|  |  | 0 | 45.9 (39.8, 52.9) | 42.5 (36.4, 49.7) | 64.3 (55.3, 74.8) | 63.9 (54.0, 75.5) | 74.3 (64.9, 85.0) | 50.4 (43.2, 58.6) | 178 (157, 203) | 139 (121, 160) |
|  |  | 21 | 218 (193, 247) | 220 (188, 256) | 366 (326, 411) | 350 (308, 398) | 309 (277, 345) | 263 (232, 296) | 703 (643, 769) | 601 (540, 668) |
|  | Pooled IIV3 |  | N=146 | N=129 | N=146 | N=128 | N=75 | N=63 | N=71 | N=66 |
|  |  | 0 | 42.3 (32.5, 55.1) | 46.7 (35.5, 61.6) | 79.8 (61.2, 104) | 61.7 (45.0, 84.5) | 91.5 (66.8, 125) | 43.9 (30.2, 63.9) | 168 (117, 240) | 171 (121, 243) |
|  |  | 21 | 233 (189, 288) | 312 (244, 401) | 414 (339, 505) | 405 (321, 511) | 288 (219, 377) | 318 (227, 447) | 734 (576, 935) | 660 (531, 821) |

Values are for all participants vaccinated with available pre- and post-vaccination HAI titers. Abbreviations: CI, confidence interval; GMT, geometric mean titer; HAI, hemagglutination inhibition; IIV3, trivalent inactivated influenza vaccine; IIV4, quadrivalent inactivated influenza vaccine.

^a^ Participants were considered at risk if they had chronic respiratory, heart, renal, metabolic, or hematological disorders.

**Supplemental Table S2. Proportions of younger and older adult participants reporting solicited reactions**

|  |  | **% (95% CI)** | | | |
| --- | --- | --- | --- | --- | --- |
|  |  | **18-60 years** | | **>60 years** | |
|  |  | **Pooled IIV4** | **Pooled IIV3** | **Pooled IIV4** | **Pooled IIV3** |
| **Solicited reaction** | **Grade** | **N=833** | **N=278** | **N=833** | **N=276** |
| Any solicited reaction | Any | 69.4 (66.1, 72.5) | 68.3 (62.5, 73.8) | 39.3 (35.9, 42.7) | 39.5 (33.7, 45.5) |
| Any injection-site reaction | Any | 58.1 (54.7, 61.5) | 57.6 (51.5, 63.4) | 27.7 (24.7, 30.9) | 25.0 (20.0, 30.5) |
| Injection-site pain | Any | 54.7 (51.3; 58.2) | 55.0 (49.0; 61.0) | 23.0 (20.1; 26.0)^a^ | 19.9 (15.4; 25.1) |
|  | Grade 3 | 0.4 (0.1; 1.0) | 0.4 (0.0; 2.0) | 0.1 (0.0; 0.7)^a^ | 0.0 (0.0; 1.3) |
| Injection-site erythema | Any | 9.2 (7.4; 11.4) | 5.8 (3.3; 9.2) | 7.6 (5.9; 9.6) | 6.9 (4.2; 10.5) |
|  | Grade 3 | 0.0 (0.0; 0.4) | 0.4 (0.0; 2.0) | 0.0 (0.0; 0.4) | 0.7 (0.1; 2.6) |
| Injection-site swelling | Any | 6.2 (4.7; 8.1) | 3.6 (1.7; 6.5) | 3.2 (2.1; 4.7) | 3.3 (1.5; 6.1) |
|  | Grade 3 | 0.0 (0.0; 0.4) | 0.0 (0.0; 1.3) | 0.0 (0.0; 0.4) | 0.0 (0.0; 1.3) |
| Injection Site Induration | Any | 6.5 (4.9; 8.4) | 5.4 (3.1; 8.7) | 2.6 (1.7; 4.0) | 3.3 (1.5; 6.1) |
|  | Grade 3 | 0.0 (0.0; 0.4) | 0.0 (0.0; 1.3) | 0.0 (0.0; 0.4) | 0.0 (0.0; 1.3) |
| Injection-site ecchymosis | Any | 0.7 (0.3; 1.6) | 0.7 (0.1; 2.6) | 0.5 (0.1; 1.2) | 0.4 (0.0; 2.0) |
|  | Grade 3 | 0.0 (0.0; 0.4) | 0.0 (0.0; 1.3) | 0.0 (0.0; 0.4) | 0.0 (0.0; 1.3) |
|  |  |  |  |  |  |
| Any systemic | Any | 42.5 (39.1; 45.9) | 40.6 (34.8; 46.7) | 24.4 (21.5; 27.4) | 23.9 (19.0; 29.4) |
| Fever | Any | 0.7 (0.3; 1.6)^a^ | 0.4 (0.0; 2.0) | 1.1 (0.5; 2.1)^b^ | 0.4 (0.0; 2.0) |
|  | Grade 3 | 0.0 (0.0; 0.4)^a^ | 0.0 (0.0; 1.3) | 0.2 (0.0; 0.9)^b^ | 0.0 (0.0; 1.3) |
| Headache | Any | 26.7 (23.7; 29.8) | 27.0 (21.9; 32.6) | 15.2 (12.9; 17.9) | 12.0 (8.4; 16.4) |
|  | Grade 3 | 1.7 (0.9; 2.8) | 1.1 (0.2; 3.1) | 0.5 (0.1; 1.2) | 0.4 (0.0; 2.0) |
| Malaise | Any | 18.5 (15.9; 21.3) | 21.6 (16.9; 26.9) | 10.4 (8.5; 12.7) | 11.2 (7.8; 15.6) |
|  | Grade 3 | 1.4 (0.7; 2.5) | 0.7 (0.1; 2.6) | 0.6 (0.2; 1.4) | 0.7 (0.1; 2.6) |
| Myalgia | Any | 26.3 (23.3; 29.4) | 20.9 (16.2; 26.1) | 12.8 (10.6; 15.3) | 11.6 (8.1; 16.0) |
|  | Grade 3 | 0.5 (0.1; 1.2) | 0.7 (0.1; 2.6) | 0.6 (0.2; 1.4) | 0.0 (0.0; 1.3) |
| Shivering | Any | 7.6 (5.9; 9.6) | 6.1 (3.6; 9.6) | 4.4 (3.1; 6.1) | 4.0 (2.0; 7.0) |
|  | Grade 3 | 0.6 (0.2; 1.4) | 0.0 (0.0; 1.3) | 0.4 (0.1; 1.0) | 0.4 (0.0; 2.0) |

Values are for all participants vaccinated. Abbreviations: CI, confidence interval; IIV3, trivalent inactivated influenza vaccine; IIV4, quadrivalent inactivated influenza vaccine.

^a^ N=832

^b^ N=827

**Supplemental Table S3. Unsolicited AEs**

|  | **Pooled IIV4s** | | **Pooled IIV3s** | |
| --- | --- | --- | --- | --- |
|  | **N=1668** | | **N=554** | |
| **Event** | **n** | **% (95% CI)** | **n** | **% (95% CI)** |
| Immediate AE (<30 min) |  |  |  |  |
| Any | 1 | <0.1 (0.0, 0.3) | 2 | 0.4 (0.0, 1.3) |
| Vaccine-related | 0 | 0.0 (0.0, 0.2) | 1 | 0.2 (0.0, 1.0) |
| Unsolicited AE within 21 days |  |  |  |  |
| Any | 303 | 18.2 (16.3, 20.1) | 99 | 17.9 (14.8, 21.3) |
| Vaccine-related | 78 | 4.7 (3.7, 5.8) | 20 | 3.6 (2.2, 5.5) |
| SAE within 6 months |  |  |  |  |
| Any | 45 | 2.7 (2.0, 3.6) | 15 | 2.7 (1.5, 4.4) |
| Vaccine-related | 0 | 0.0 (0.0, 0.2) | 0 | 0.2 (0.0, 1.0) |
| Death within 6 months | 3 | 0.2 (0.0, 0.5) | 0 | 0.0 (0.0, 0.7) |

Values are for all participants vaccinated. Abbreviations: AE, adverse event; CI, confidence interval; IIV3, trivalent inactivated influenza vaccine; IIV4, quadrivalent inactivated influenza vaccine; SAE, serious adverse event.
